# Characterising the aetiology of severe acute gastroenteritis among patients visiting a hospital in Qatar using real-time polymerase chain reaction

**DOI:** 10.1186/1471-2334-13-329

**Published:** 2013-07-18

**Authors:** Asma Al-Thani, Maide Baris, Najah Al-Lawati, Said Al-Dhahry

**Affiliations:** 1 Qatar University, P.O. BOX 2713, Doha, Qatar; 2 Laboratory Medicine Department, Hamad Medical Corporation, Doha, Qatar

**Keywords:** Norovirus, Rotavirus, Adenovirus, Astrovirus, Acute gastroenteritis

## Abstract

**Background:**

Acute gastroenteritis (AGE) remains a common cause of clinic visits and hospitalizations, though its aetiology has not been determined in Qatar.

**Methods:**

We performed a prospective, emergency department–based study of 288 children and adults with AGE. Stool specimens were collected at presentation from June to November 2009. Faecal specimens were tested, using real-time PCR, for a panel of four viral (norovirus, adenovirus, astrovirus and rotavirus) and bacterial pathogens.

**Results:**

Viral and bacterial pathogens were detected in 131 (45.5%) and 34 (12.2%) of the 288 patients recruited. The most commonly detected pathogens were norovirus (28.5%), rotavirus (10.4%), followed by adenovirus (6.25%) and astrovirus (0.30%). Norovirus was the most commonly detected viral pathogen amongst all the age groups with an almost even distribution in all age groups. Rotavirus and adenovirus were more common in children under 5 yr of age. Astrovirus was found in only one person.

**Conclusions:**

Viruses, especially noroviruses, are associated with severe diarrhoea in children and adults in Qatar. Further studies to confirm the findings and to explore the causes of illness among patients from whom a pathogen cannot be determined are needed.

## Background

Acute gastroenteritis (AGE) is a common cause for hospital/clinic visits and hospitalisations in children as well as adults [1,2]. It is characterised by diarrhoea, vomiting and abdominal pain that may lead to hypovolemic shock and dehydration, and subsequently death in severe cases. Children in developing countries are particularly at risk of both morbidity and mortality [1]. Worldwide, gastroenteritis affects 3 to 5 billion children each year, and accounts for 1.5 to 2.5 million deaths per year or 12% of all deaths among children less than 5 years of age [1]. In developed countries AGE accounts for 300 deaths per year [1].

Among adults, an estimated 179 million cases of AGE occur each year, resulting in millions of clinic visits, nearly 500,000 hospitalisations, and >5000 deaths [2]. Although the burden of AGE in children has been described extensively, there is little data on AGE in adults.

Four major viral pathogens have been associated with AGE. These include three RNA viruses (rotavirus, norovirus, and astrovirus) and one DNA virus (enteric adenovirus) [3,4]. Saporovirus, Aichivirus and members of enterovirus group also cause AGE but are less common [5].

Noroviruses are now recognised as the leading cause of epidemic gastroenteritis in all age groups, accounting for over 90% of viral gastroenteritis outbreaks and approximately 50% of all gastroenteritis outbreaks [6]. Past reviews have shown that norovirus was responsible for 4.4%–8.7% of AGE cases among adults and elderly individuals admitted to the emergency department (ED) or hospitalized [7]. In Bangladesh, it is estimated that 200,000 deaths are caused by norovirus gastroenteritis annually though more data on viral aetiology in AGE in developing countries is sparse [8]. Rotavirus is the most common cause of severe childhood diarrhoea worldwide [9]. It has seven serogroups (A-G) that are classified on the basis of antigenic variations. Each year worldwide, rotavirus causes approximately 111 million episodes of gastroenteritis requiring only home care, 25 million office visits, and 2 million hospitalizations [10]. Enteric adenoviruses can also cause AGE. The prevalence rate of adenovirus gastroenteritis is not as high as rotavirus or norovirus infections, however it is still very important to monitor these infections for epidemiologic and surveillance studies. Enteric adenovirus have been related to AGE in variable frequency (ranging from 1.4 to 10%), depending on the geographic settings [11,12]. Astrovirus has also been associated with AGE, with a frequency range between 2% to 26% [13]. However, there is a paucity of data on viral aetiology in AGE in the Middle East both in adult and in paediatric population. Indeed, in Qatar there has not been any study investigating viral aetiology of AGE to date.

This study prospectively enrolled and tested children and adults with AGE presenting to our hospital to determine the frequency and aetiology of viral infectious AGE, using real-time reverse transcription (RT)-PCR and primers for adenovirus, astrovirus, enterovirus, and rotavirus. To our knowledge, no study has reported complete information regarding the number and distribution of these four viruses responsible for gastroenteritis in hospitalised patients (children and adults) in Qatar specifically or in the Middle East in general.

## Methods

### Patients

Patients who visited the hospital at Hamad Medical Corporation (HMC), Qatar with AGE-like complaints were enrolled in the study; they were aged 1 month to 84 yr old. Demographic information and clinical history was collected for all the patients. A total of 288 patients were enrolled in the study. The patients were enrolled in the study between June 2009 and November 2009.

Patients who visited the hospital with complaints of AGE were enrolled in the study after excluding those with bloody and formed stools. AGE was defined as ≥1 episode diarrhoea with abdominal discomfort with 24 hour period and passing of loose/watery/soft stool.

All enrolled patients/their guardians, in case of children gave written informed consent. The study was approved by the ethical board at Hamad Medical Research Center.

### Stool samples collection and pre-treatment

Stool samples were collected from each subject during the hospital visit within 24 hours. The samples were obtained only once from each patient. A total of 288 samples were collected (one sample per patient) and frozen at −86°C. The frozen samples were transported to Qatar University Health Science Department research laboratory for analysis.

One hundred milligram (100 mg) of solid or liquid stool was re-suspended in 900 μl Buffer ASL (Stool Lysis Buffer). Each sample was then vortexed vigorously for 1 minute. Samples were then incubated for 10 minutes at room temperature to allow sedimentation of large stool particles. Approximately 500 μl supernatant was transferred from the top of the suspension to a fresh screw cap tube without carryover of large stool particles. Finally, samples were incubated for 10 minutes at 70°C in a thermoshaker.

### Extraction of viral RNA

Pre-treated samples were placed into an extraction instrument, The EZ1 Mini. The manufacturer’s instructions were followed to extract viral RNA (QIAGEN QIAamp Viral RNA Mini Kits EZ1 Virus Mini Kit v2.0, catalog number 955134, Germany).

#### Viruses

Amplification and detection of the four viruses (norovirus, adenovirus, rotavirus and astrovirus) was performed by a two tube multiplex PCR assay (Fast Track Diagnostics, Luxemburg) using an ABI 7500 Real Time PCR instrument, according to the manufacturer’s instructions.

#### Bacteria

A swab of each stool specimen was examined for bacteria, including *Salmonella spp., Shigella spp., E. coli spp,* and *Campylobacter spp* using routine diagnostic assays.

### Statistical analysis

Prevalence rates were expressed as percentages. No statistical analyses were performed for this study.

## Results

### Patients

During the study period, 288 subjects were enrolled. Patient demographics are summarised in Table 1.

**Table 1:** Patient characteristics

**Characteristic**	**Value (n = 288)**	
Age, years	16.6 (≤1 -84)	
Median (range)	38 (13.2%)	
<1	83 (29%)	
1-10	29 (10.1%)	
11-20	75 (26%)	
21-50	24 (8.3%)	
51-60	29 (10.1%)	
>60	10 (3.5%)	
Unspecified age		
Gender		
Male	153 (53.1%)	
Female	129 (44.8%)	
Sex not recorded	6 (2.1%)	
Chronic diseases	Total = 27 (9.4%)	
	Cardiovascular disease (CVD)	2
	Acute renal failure	1
	Renal disease	8
	Anaemia	11
	Lymphoma/Leukemia	2
	Liver disease	2
	Irritable bowel syndrome	1

All the patients were Qatar residents at time of the study, though not all were Qatari nationals.

### Aetiology from the stool samples

All the subjects provided stool specimens. A total of 288 stool samples were analysed, and viral agents were identified in 131 patients (45.5%), followed by bacterial infections (n = 34; 12.2%).

Table 2 summarises the sample numbers which tested positive for virus types by month of study. The highest number of infections was detected in July and September (the warm season in Qatar).

**Table 2:** Samples tested positive for viruses by month

**Month**	**Viruses N (%)**			
	**Norovirus**	**Rotavirus**	**Adenovirus**	**Astrovirus**
N = 281*	**N = 82**	**N = 30**	**N = 18**	**N = 1**
June (n = 37)	10 (27%)	8 (22%)	2 (5%)	0
July (n = 99)	32 (32%)	11 (11%)	8 (8%)	1 (1%)
August (n = 32)	15 (47%)	1 (3%)	1 (3%)	0
September (n = 72)	16 (22%)	10 (14%)	5 (7%)	0
October (n = 41)	9 (22%)	0	2 (5%)	0

*Seven samples were excluded due to poor sample quality or due to not obtaining enough samples to analyse.

Overall, norovirus was detected in 28.5% of stool specimens (82 of 288); rotavirus was detected in 10.4% (30 of 288); adenovirus was detected in 6.25% (18 out of 288); and astrovirus was detected in 0.30% (1 out of 288). No patients were co-infected with more than one virus. Norovirus was the predominant viral agent identified in this study.

*Salmonella* was the most common bacterial species detected (n = 23), followed by *E. Coli* (n = 6); *Shigella* (n = 3); and *Campylobacter* (n = 3).

### Epidemiologic features

The distribution of patients according to age is shown in Table 3.

**Table 3:** Viral aetiology distribution by patient age

**Age (yr)**	**Norovirus (N = 82)**	**Rotavirus (N = 30)**	**Adenovirus (N = 18)**	**Astrovirus (N = 1)**
<1	10 (12.2%)	11 (36.7%)	3 (16.7%)	0
N = 38				
1-10	17 (20.7%)	10 (33.3%)	8 (44.4%)	0
N = 83				
11-20	9 (11.0%)	0	2 (11.1%)	1 (100%)
N = 29				
21-50	29 (35.4%)	5 (16.7%)	2 (11.1%)	0
N = 75				
51-60	7 (8.5%)	4 (13.3%)	0	0
N = 24				
>60	10 (12.2%)	0	3 (16.7%)	0
N = 29				
Unspecified age	0	0	0	0
N = 10				
**Total**	**82 (28.4**%**)**	**30 (10.4**%**)**	**18 (6.25)**	**1 (0.30**%**)**

Norovirus was the most commonly detected viral pathogen amongst all the age groups with an almost even distribution in all age groups. Rotavirus and adenovirus were more common in children under 5 yr. However, the numbers were too small to make definitive conclusions. Astrovirus was found in only one person.

## Discussion

This study provides novel data on the infectious causes of AGE in children and adults in Qatar. A viral agent was identified in almost 50% of the patients (131/288). No correlations of symptom severity and viral aetiology were made in this study. The causes of these diseases (e.g. recent travel, food, etc.) were not investigated. Although the highest rate of infection was observed in July and September, which constitute the warm season in Qatar, it is difficult to make accurate conclusions re seasonality due to the low number of the samples; the fact that the samples were tested only once during the 6 month study period and the differences could be explained by the higher number of subjects recruited during those two months (Table 2).

Norovirus was the most frequently identified cause of hospital visits for AGE, with detection in 28.4% and an almost even distribution across all age groups. This correlates well with a study of AGE in adults by Bresee *et al.*, who also found norovirus to be the most common aetiologic agent for AGE (detected in 27% of patients with a median age of 31 yr) [2]. Furthermore, other studies on adults in industrialised countries have also identified norovirus as the leading cause of AGE – infecting 30% of adults in England [14-16]. Noroviruses has been detected in 16% of cases and 3% of controls in outpatient clinics in Germany across all age groups [17]. However, other studies have found a prevalence ≤10% for norovirus in AGE [17-19]. The higher prevalence in our study may reflect a higher rate of infection with norovirus in Qatar though larger studies may be required to corroborate this finding.

Rotavirus was found in 10.4% of subjects, mostly children less than 5 years of age. This is consistent with previous studies which have shown that rotaviruses are the most common cause of severe gastroenteritis among young children, but adult cases are thought to be uncommon [20-22]. The nine cases of rotavirus infection in adults may reflect the lack of rotavirus children’s immunisation programme in Qatar until very recently (March 2009). The vaccine is now offered to all children less than 2 years of age, therefore, many patients would not have been immunised when this study was undertaken. Rotavirus vaccine may also confer protection in parents of vaccinated children [23]. Thus, conducting such a study in the rotavirus vaccine era is a limitation in itself.

*Salmonella* and *E. Coli* species were the most common bacterial pathogens identified. However, antiobiotic use was not monitored in this study therefore it is difficult to comment on these findings.

This study has several limitations. This study did not take into account correlation of clinical symptoms or duration of illness with viral aetiology. Therefore, we cannot ascertain variables that would help clinicians target specific patients for testing. Furthermore, our samples were collected from June 2009 to November 2009, which are mostly the summer months in Qatar and many residents travel to various countries and may carry viruses back with them though no data had been collected to assess patients for recent international travel; therefore, the contribution of this factor to viral aetiology is not known. In addition, we investigated only one strain of adenovirus, astrovirus and rotavirus; and two strains of norovirus. All common strains of these viruses should be investigated in future studies. Moreover, only patients presenting to a hospital were included in this study. In order to investigate the true burden of viral gastroenteritis, community-based studies are necessary. Finally, the lack of healthy controls limits conclusions about the relative importance of each pathogen. Despite these limitations, this study provides the most comprehensive data to date on the aetiology of AGE in patients in Qatar.

This study indicated that the two most common gastroenteritis viruses in Qatar are the norovirus and rotavirus. Nevertheless, large scale controlled studies are necessary to fully understand the aetiology and burden of viral pathogens in AGE in Qatar. Future studies should be carried out in samples collected throughout the year to assess the impact of winter vs. summer infections and international travel. Correlations of viruses with clinical symptoms and symptom severity also need to be performed.

## Conclusion

The epidemiologic surveillance of enteric viruses is a key step in understanding the burden of these infections in human health and their impact on the national health costs in Qatar. To date, no data on viral epidemiologic surveillance of AGE have been published in Qatar. The high prevalence of viral agents found in this study may have important implications for testing patients with AGE and minimizing use of antibiotics. The aim of control and prevention plans for viral gastroenteritis is to minimise nosocomial infections, to limit unnecessary antibiotic treatment, and ultimately to help develop a vaccination/immunisation campaign at the national level, if necessary, which are all significant improvements to the health system needed in Qatar.

## Competing interests

The authors declare that they have no competing interests.

## Authors’ contributions

AA-T and SA-D developed the study design and applied for research grants to fund the study. MB and NA-L collected samples from the patients, conducted the study and performed data analysis. AA-T and MB wrote the final publication and submitted it. All authors read and approved the final manuscript.

## Pre-publication history

The pre-publication history for this paper can be accessed here:

http://www.biomedcentral.com/1471-2334/13/329/prepub

## References

[B1] Chow CM Leung AK Hon KL Acute gastroenteritis: from guidelines to real life Clin Exp Gastroenterol 2010 3 97 112 10.1007/s12328-010-0138-0 21694853 PMC3108653

[B2] Bresee JS Marcus R Venezia RA Keene WE Morse D Thanassi M Brunett P Bulens S Beard RS Dauphin LA Slutsker L Bopp C Eberhard M Hall A Vinje J Monroe SS Glass RI US Acute Gastroenteritis Etiology Study Team The etiology of severe acute gastroenteritis among adults visiting emergency departments in the United States J Infect Dis 2012 205 1374 1381 10.1093/infdis/jis206 22454468

[B3] Clark B McKendrick M A review of viral gastroenteritis Curr Opin Infect Dis 2004 17 461 469 10.1097/00001432-200410000-00011 15353966

[B4] Wood DJ Adenovirus gastroenteritis Br Med J (Clin Res Ed) 1988 296 6617 229 230 10.1136/bmj.296.6617.229-a PMC2544764 2829987

[B5] Monroe SS Control and prevention of viral gastroenteritis Emerg Infect Dis 2011 17 8 1347 1348 21801608 10.3201/eid1708.110824 PMC3381538

[B6] Patel MM Hall AJ Vinje J Parashar UD Noroviruses: a comprehensive review J Clin Virol 2009 44 1 8 10.1016/j.jcv.2008.10.009 19084472

[B7] Haustein T Harris JP Pebody R Lopman BA Hospital admissions due to norovirus in adult and elderly patients in England Clin Infect Dis 2009 49 1890 1892 10.1086/648440 19911997

[B8] Rahman M Hassan Z Nahar Z Faruque AS Van Ranst M Rahman SR Azim T Molecular detection of noroviruses in hospitalized patients in Bangladesh Eur J Clin Microbiol Infect Dis 2010 29 937 945 10.1007/s10096-010-0948-5 20467770

[B9] Khoury H Ogilvie I El Khoury AC Duan Y Goetghebeur MM Burden of rotavirus gastroenteritis in the Middle Eastern and North African pediatric population BMC Infect Dis 2011 11 9 10.1186/1471-2334-11-9 21214934 PMC3022719

[B10] Parashar UD Hummelman EG Bresee JS Miller MA Glass RI Global illness and deaths caused by rotavirus disease in children Emerg Infect Dis 2003 9 565 572 10.3201/eid0905.020562 12737740 PMC2972763

[B11] Ramani S Kang G Viruses causing childhood diarrhoea in the developing world Curr Opin Infect Dis 2009 22 477 482 10.1097/QCO.0b013e328330662f 19633550

[B12] Tran A Talmud D Lejeune B Jovenin N Renois F Payan C Leveque N Andreoletti L Prevalence of rotavirus, adenovirus, norovirus, and astrovirus infections and coinfections among hospitalized children in Northern France J Clin Microbiol 2010 48 1943 1946 10.1128/JCM.02181-09 20305010 PMC2863921

[B13] Li CY Liu N Guo WD Yu Q Wang WR Song ZZ Yan H Luo Y Lu AT Li HY Zhu L Duan ZJ Outbreak of neonatal gastroenteritis associated with astrovirus serotype 1 at a hospital in Inner Mongollia, China J Clin Microbiol 2010 48 4306 4309 10.1128/JCM.00797-10 20810774 PMC3020857

[B14] Amar CF East CL Gray J Iturriza-Gomara M Maclure EA McLauchlin J Detection by PCR of eight groups of enteric pathogens in 4,627 faecal samples: re-examination of the English case–control Infectious Intestinal Disease Study (1993–1996) Eur J Clin Microbiol Infect Dis 2007 26 311 323 10.1007/s10096-007-0290-8 17447091

[B15] Karsten C Baumgarte S Friedrich AW von Eiff C Becker K Wosniok W Ammon A Bockemühl J Karch H Huppertz HI Incidence and risk factors for community-acquired acute gastroenteritis in north-west Germany in 2004 Eur J Clin Microbiol Infect Dis 2009 28 935 943 10.1007/s10096-009-0729-1 19319582 PMC2723666

[B16] Huhulescu S Kiss R Brettlecker M Cerny RJ Hess C Wewalka G Allerberger F Etiology of acute gastroenteritis in three sentinel general practices, Austria 2007 Infection 2009 37 103 108 10.1007/s15010-008-8106-z 19148576

[B17] de Wit MA Koopmans MP Kortbeek LM Wannet WJ Vinjé J van Leusden F Bartelds AI van Duynhoven YT Sensor, a population-based cohort study on gastroenteritis in the Netherlands: incidence and etiology Am J Epidemiol 2001 154 666 674 10.1093/aje/154.7.666 11581101

[B18] de Wit MA Koopmans MP Kortbeek LM van Leeuwen NJ Vinje J van Duynhoven YT Etiology of gastroenteritis in sentinel general practices in The Netherlands Clin Infect Dis 2001 33 280 288 10.1086/321875 11438890

[B19] Rockx B De Wit M Vennema H Vinjé J De Bruin E Van Duynhoven Y Koopmans M Natural history of human calicivirus infection: a prospective cohort study Clin Infect Dis 2002 35 246 253 10.1086/341408 12115089

[B20] Wolfaardt M Taylor MB Booysen HF Engelbrecht L Grabow WO Jiang X Incidence of human calicivirus and rotavirus infection in patients with gastroenteritis in South Africa J Med Virol 1997 51 290 296 10.1002/(SICI)1096-9071(199704)51:4<290::AID-JMV6>3.0.CO;2-0 9093943

[B21] Staat MA Azimi PH Berke T Roberts N Bernstein DI Ward RL Pickering LK Matson DO Clinical presentations of rotavirus infection among hospitalized children Pediatr Infect Dis J 2002 21 221 227 10.1097/00006454-200203000-00012 12005086

[B22] de Wit MA Koopmans MP Kortbeek LM van Leeuwen NJ Bartelds AI van Duynhoven YT Gastroenteritis in sentinel general practices, The Netherlands Emerg Infect Dis 2001 7 82 91 10.3201/eid0701.010113 11266298 PMC2631671

[B23] Cortese MM Tate JE Simonsen L Edelman L Parashar UD Reduction in gastroenteritis in United States children and correlation with early rotavirus vaccine uptake from national medical claims databases Pediatr Infect Dis J 2010 29 489 494 20354464 10.1097/INF.0b013e3181d95b53

